# Genetic Transformation and Genomic Resources for Next-Generation Precise Genome Engineering in Vegetable Crops

**DOI:** 10.3389/fpls.2017.00241

**Published:** 2017-02-22

**Authors:** Teodoro Cardi, Nunzio D’Agostino, Pasquale Tripodi

**Affiliations:** Consiglio per la ricerca in agricoltura e l’analisi dell’economia agraria (CREA), Centro di ricerca per l’orticoltura, Pontecagnano FaianoItaly

**Keywords:** vegetable crops, genome editing, *in vitro* regeneration, genetic transformation, whole genome sequences, genomics, breeding

## Abstract

In the frame of modern agriculture facing the predicted increase of population and general environmental changes, the securement of high quality food remains a major challenge to deal with. Vegetable crops include a large number of species, characterized by multiple geographical origins, large genetic variability and diverse reproductive features. Due to their nutritional value, they have an important place in human diet. In recent years, many crop genomes have been sequenced permitting the identification of genes and superior alleles associated with desirable traits. Furthermore, innovative biotechnological approaches allow to take a step forward towards the development of new improved cultivars harboring precise genome modifications. Sequence-based knowledge coupled with advanced biotechnologies is supporting the widespread application of new plant breeding techniques to enhance the success in modification and transfer of useful alleles into target varieties. Clustered regularly interspaced short palindromic repeats (CRISPR)/CRISPR-associated protein 9 system, zinc-finger nucleases, and transcription activator-like effector nucleases represent the main methods available for plant genome engineering through targeted modifications. Such technologies, however, require efficient transformation protocols as well as extensive genomic resources and accurate knowledge before they can be efficiently exploited in practical breeding programs. In this review, we revise the state of the art in relation to availability of such scientific and technological resources in various groups of vegetables, describe genome editing results obtained so far and discuss the implications for future applications.

## Introduction

Vegetable crops include a large number of species belonging to various families, characterized by multiple geographical origins, large genetic variability and diverse reproductive features. As result of natural and artificial selection, various vegetables are differentially used worldwide for many purposes, either as fresh or processed products. Due to their nutritional value, vegetables have an important place in human diet, providing, in combination with freshness and taste, protection against various non-transmissible diseases and reduction of the so-called “hidden hunger” especially in developing countries (Desjardins, 2014).

Fast changes in climate and consumers’ needs as well as the appearance of emerging and re-emerging plant pests and diseases require the continuous development of novel genotypes adapted to varying horticultural systems (Anderson et al., 2004). The availability of genetic resources plays an important role in such a process. Indeed, a large number of crop wild relatives and local traditional varieties is conserved *in situ* and *ex situ* by private or public institutions. In addition, local vegetable varieties (landraces) are still grown and reproduced by farmers, based on their adaptation to specific environments and on the presence of valuable traits. New genomics and phenomics tools allow the throughout characterization of such resources and should facilitate their better use in breeding. Nonetheless, the overall level of biodiversity in horticultural systems is decreasing, with threats to their economic, social, and environmental sustainability (Maggioni, 2004; Silva Dias, 2014).

**FIGURE 1:**
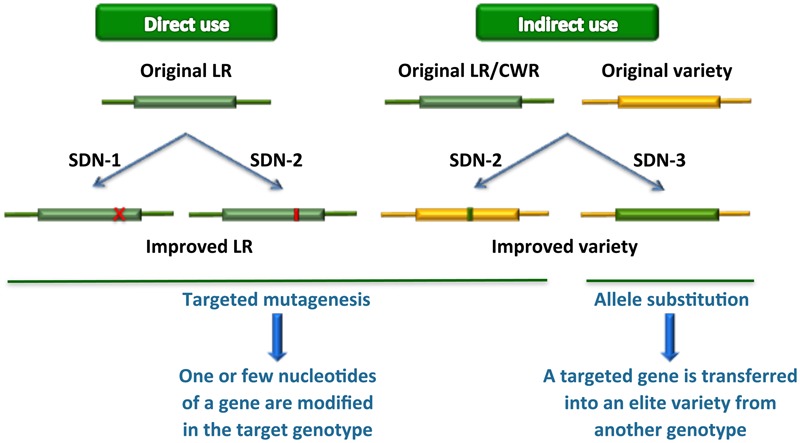
**Novel plant breeding techniques applied to the exploitation of genetic resources.** LR, landraces; CWR, crop wild relatives. SDN-1, 2, 3, site-directed nuclease usage for gene knockout, gene editing or gene replacement/stacking, respectively. If the gene replaced by the latter approach derives from a sexually compatible genetic resource, it can be dubbed also as “cisgenesis at the same locus.”

New plant breeding techniques, in particular gene transfer based on cisgenic approaches and next-generation precision genome engineering relying on genome editing technologies, can play a key role in accessing genetic resources and using them in functional studies and streamlined breeding strategies (Cardi, 2016) (**Figure 1**). Such approaches, however, require efficient transformation protocols as well as extensive genomic resources and accurate knowledge before they can be efficiently exploited in practical breeding programs. In this review, we revise the state of the art in relation to availability of such scientific and technological resources in various groups of vegetables, describe genome editing results obtained so far and discuss the implications for future applications.

## Regeneration and Transformation

Available plant transformation methods include indirect (i.e., requiring an intermediate biological vector, usually the bacterium *Agrobacterium tumefaciens*) and direct methods (electroporation or PEG-mediated transformation of protoplasts, biolistics, etc.; Finer, 2010). Successful transformation, however, relies on various phases, being the introduction and integration of DNA into the plant genome as well as the selection and regeneration of transformed cells the most important and, sometimes, limiting factors (Wȩdzony et al., 2014; Altpeter et al., 2016). Regeneration of transformed plants has been extensively pursued in vegetable crops, although large differences in efficiency among families, species, and cultivars have been reported (Klocke et al., 2010). Plant regeneration is generally achieved via *in vitro* culture systems, using a range of explants and following two alternative pathways: *de novo* shoot organogenesis (DNSO) or somatic embryogenesis (SE; Wȩdzony et al., 2014).

Within the Solanaceae family, eggplant (*Solanum melongena*) and, to a lesser extent, tomato (*S. lycopersicum*) have been successfully subjected to genetic transformation with various purposes using different approaches (Klocke et al., 2010). Regeneration in eggplant has been obtained either through SE or DNSO starting from protoplasts, tissue explants, microspores (Rotino et al., 2014). In tomato, Agrobacterium-mediated transformation of cotyledon explants has been most frequently achieved, albeit protoplast protocols are also available. Genetic variability of response to both *in vitro* systems is well known (Kurtz and Lineberger, 1983; Derks, 1992). The dominant *Rg1* gene involved in regeneration capacity has been transferred from *S. peruvianum* into *S. lycopersicum* and subsequently mapped on chromosome 3 (Koornneef et al., 1993). Recently, its effect has been characterized in a different genetic background also in combination with other relevant genes (Pino et al., 2010; Lombardi-Crestana et al., 2012). By contrast, although some positive results have been reported (Klocke et al., 2010), pepper (*Capsicum* spp.) is considered a recalcitrant species due to several issues that jeopardize the *in vitro* response (Kothari et al., 2010). Some interesting results, however, based on the over-expression of two heterologous transcription factors involved in the regeneration process (i.e., WUSCHEL and BABY BOOM), have been recently published in sweet and chili pepper (Solís-Ramos et al., 2009; Heidmann et al., 2011).

*Brassica oleracea* and *B. rapa* include the majority of vegetables belonging to Brassicaceae. Transformation, usually achieved by inoculation of seedling explants with Agrobacterium (direct gene transfer using protoplasts has been also accomplished) followed by regeneration through organogenesis, is strongly dependent on the genotype and various other factors, being *B. rapa* genotypes more difficult to transform than *B. oleracea* ones, and cabbage (*B. oleracea* var. *capitata*) the most difficult type within the latter. A strong genetic component with significant additive effects both for *A. tumefaciens* susceptibility and regeneration ability from cotiledonary explants as well as from leaf protoplasts has been found in *B. oleracea* [(Sparrow et al., 2004) and references cited therein], suggesting the possibility to incorporate those traits in recalcitrant genotypes by transferring the relevant genes. *Raphanus sativus*, another vegetable of the same family, is considered recalcitrant and only few reports are available. Advances in regeneration and transformation in this group of crops have been reviewed elsewhere (Vinterhalter et al., 2007; Klocke et al., 2010; Sparrow et al., 2011; Park et al., 2012; Kumar and Srivastava, 2016). With the aim to avoid tissue culture systems, similarly to the related species *Arabidopsis thaliana, in planta* transformation systems based on floral dipping or flower bud microinjection have been also attempted in various genotypes (Curtis and Nam, 2001; Sparrow et al., 2011).

Within the Cucurbitaceae family, transformation has been accomplished in all the three genera including vegetable crops: *Cucumis* (cucumber and melon), *Cucurbita* (squash, pumpkin and zucchini), and *Citrullus* (watermelon). Various (young) explants have been generally inoculated with Agrobacterium followed by regeneration through shoot organogenesis, but transformation efficiency varied largely with the genotype used (Klocke et al., 2010; Manamohan et al., 2011). Some improvements in the protocol have been lately reported for melon (Bezirganoglu et al., 2014; Zhang et al., 2014), cucumber (Wang et al., 2015), *Cucurbita* spp. (Nanasato et al., 2013), and watermelon (Liu et al., 2016). In the latter species, an efficient regeneration procedure *via* SE from embryogenic calli derived from leaf explants has been also recently published (Vinoth and Ravindhran, 2015). Attempts to develop *in planta* methods (either *via* pollen tube or microinjection of the shoot apical meristem) have been made in watermelon and cucumber (Chen et al., 1998; Baskaran et al., 2016). As far as genetic and molecular aspects of shoot regeneration are concerned, a simple dominant control of regeneration ability from leaf explants has been found in cucumber (Nadolska-Orczyk and Malepszy, 1989), while distinct expression profiles of WUSCHEL-related homeobox (WOX) genes have been associated with different regeneration abilities in watermelon (Zhang N. et al., 2015).

Among Asteraceae, lettuce (*Lactuca sativa*) and chicory (*Cichorium intybus*) have been largely and successfully used in many transformation experiments for a variety of purposes, using either Agrobacterium inoculation of various explants (mainly cotyledons and true leaves) or direct gene transfer (electroporation/PEG treatment of protoplasts or particle bombardment of tissue explants) (Davey et al., 2007; Klocke et al., 2010; Song et al., 2014; Matvieieva, 2015). Shoot regeneration from hairy roots has also been accomplished. Regeneration normally proceeds by organogenesis, but SE has also been reported. Recently, the “surface response” method has been employed in lettuce to optimize plant regeneration (Gómez-Montes et al., 2015). While a mature regeneration/transformation technology is available for both above-mentioned species, *de novo* shoot regeneration and cell transformation have been observed only sporadically and with very low efficiency in another important vegetable of this family, i.e., globe artichoke (*Cynara cardunculus* var. *scolymus*; Menin et al., 2012).

Carrot (*Daucus carota*), a member of the Apiaceae family, is considered a model species for *in vitro* SE. Factors affecting transformation efficiency have been defined in the late 1980 – early 1990. Subsequently, genetic transformation has been pursued for various objectives using either indirect (*A. tumefaciens* and *A. rhizogenes*) or direct gene transfer methods (electroporation/PEG-mediated transformation of protoplasts and biolistics) (Punja et al., 2007; Baranski, 2008; Klocke et al., 2010). Much less work has been performed in other vegetables of the same family, such as fennel (*Foeniculum vulgare*), celery (*Apium graveolens*), and parsley (*Petroselinum crispum*; Baranski, 2008; Klocke et al., 2010). Fennel is able to regenerate *in vitro* by either organogenesis or embryogenesis, albeit with a marked genotypic effect (Anzidei et al., 2000; Jakhar and Choudhary, 2012; Saxena et al., 2012), but no transgenic plants have been generated. A reproducible protocol has been developed for rapid and efficient production of transgenic celery plants *via* somatic embryo regeneration from *A. tumefaciens*-inoculated leaf sections, cotyledons, and hypocotyls (Song et al., 2007). Only transgenic calli have been obtained in parsley (Baranski, 2008).

Successful Agrobacterium-mediated transformation in the leafy vegetable *Spinacia oleracea* (Chenopodiaceae) has been first published in 1995 and later applied for transferring agronomically relevant genes (Klocke et al., 2010). In the meantime, efforts have been made to improve transformation and regeneration procedures. Although the genotype has generally shown a significant effect, regeneration has been achieved either through organogenesis or embryogenesis depending mainly on the explant type and auxin/cytokinin ratio: cotyledon explants and low ratios facilitate the former, whereas root explants and high ratios favor the latter. In addition, low temperature (14°C), photoperiod, light intensity, and GA_3_ content had substantial effects on shoot regeneration (Geekiyanage et al., 2006; Chin et al., 2009; Nguyen et al., 2013b), while low levels of hygromycin (0.5 mg l^-1^) have been found to promote SE (Milojević et al., 2012). An optimized regeneration protocol has been recently published (Nguyen et al., 2013a).

Vegetable legumes (harvested as green immature pods and seeds or, e.g., in cowpea, also as leaves) and pulses (harvested for the dry seed) have long been considered as recalcitrant to *in vitro* transformation and regeneration, although significant progress has been shown in the recent past (Somers et al., 2003; Klocke et al., 2010; Dewir et al., 2016; Gatti et al., 2016; Nguyen et al., 2016). Difficulties in achieving organogenesis and SE from differentiated tissues have prompted the development of regeneration/transformation protocols from meristematic tissues, but this has made difficult the selection of uniform (non-chimeric) transformed shoots. In several cases, the release of phenol oxidation products from explants cultured *in vitro* inhibits cell division and provokes tissue darkening and death, while rooting of regenerated shoots can be an additional critical step. All those problems have been encountered in applying genetic transformation to common bean (*Phaseolus vulgaris*), that, compared to related species (e.g., *P. acutifolius, P. coccineus, P. polyanthus*), exhibits a lower regeneration potential. Nevertheless, various direct and indirect transformation methods have been attempted in this species, showing some interesting results (Veltcheva et al., 2005; Hnatuszko-Konka et al., 2014). The improvement of the *in vitro* selection step through the use of a systemic herbicide has resulted in a significant increase of transformation efficiency (Aragão and Campos, 2007). Recently, it has been shown that the endogenous hormonal content in embryogenic calli could be altered and SE increased by down-regulating the expression of the *PvTRX1h* gene, encoding for a histone methyltransferase (Barraza et al., 2015). Genetic engineering problems and efforts similar to those described for *P. vulgaris* have been recently reviewed in the related species *Vigna unguiculata*, for which electroporation, biolistics and Agrobacterium*-*based methods have all been used, the latter being the most common (Citadin et al., 2011). Due to the involvement of meristematic tissues in the regeneration process, also for this species it has been necessary to develop alternative selection regimes for increasing the recovery of transformed shoots (Aragão and Campos, 2007; Bakshi et al., 2012; Bakshi and Sahoo, 2013). Similarly to other legumes, also in garden pea (*Pisum sativum*), first transformed in the late 1980, meristems have been the explants of choice, but the transformation protocols have been gradually improved and successfully employed for various purposes, although a strong genotypic effect has been shown (Klocke et al., 2010; Mikschofsky and Broer, 2012). The broad bean (*Vicia faba*) is probably one of the most difficult legume species to regenerate and transform, requiring particular efforts to solve the problem of tissue blackening *in vitro* (Klenotičová et al., 2013), and only few successful experiments have been reported using Agrobacterium-mediated transformation of meristematic cells or stem segments [reviewed in (O’Sullivan and Angra, 2016)]. Due to the intrinsic difficulties of legume regeneration systems, a range of methods not requiring tissue culture of explants have been proposed (Somers et al., 2003), including the electroporation of nodal axillary buds in pea and cowpea (Chowrira et al., 1996), the Agrobacterium inoculation of germinating seeds in pea and bean (Liu et al., 2005; Svabova et al., 2005), the inoculation of flower buds in cowpea (Ilori and Pellegrineschi, 2011).

Onion, shallot, garlic, leek (Amaryllidaceae), and asparagus (Asparagaceae), as other Monocotyledons, have been initially considered recalcitrant to *in vitro* regeneration and not amenable to Agrobacterium-mediated genetic transformation. Subsequently, however, several approaches have been developed [reviewed in (Gantait et al., 2010; Klocke et al., 2010)]. In onion and shallot (*Allium cepa*), either immature zygotic embryos or young calli derived from mature embryos have been successfully used as explants in Agrobacterium-mediated transformation experiments, but the former approach can rely on a higher availability of explants during the year (Eady et al., 2000; Zheng et al., 2001). Using the zygotic embryo-derived calli, shallot genotypes gave better results than onion ones. Furthermore, to overcome some limits of both embryo-based protocols, embryogenic calli initiated from seedling radicles have been used both with Agrobacterium and biolistic systems, showing comparable results (Aswath et al., 2006). Agrobacterium and biolistic approaches have been applied for garlic (*A. sativum*) as well. Either immature zygotic embryos, similarly to onion, or calli from root segments have been transformed *via* Agrobacterium, the former approach being successful also with leek (*A. porrum*; Zheng et al., 2004; Eady et al., 2005). However, the difficulty to get seed-derived tissues has recently prompted the development of alternative methods based on immature leaf tissues (Kenel et al., 2010) or roots from *in vitro* plantlets (Ahn et al., 2013). Finally, transformation in *Asparagus officinalis* has been demonstrated as early as in the ‘90 with a range of approaches, including Agrobacterium, protoplast electroporation, and biolistics (Klocke et al., 2010), but regeneration and transformation protocols have not been developed further.

The availability of *in vitro* culture and genetic transformation protocols for various vegetables is summarized in **Table 1**.

**Table 1:** Availability of *in vitro* regeneration/genetic transformation protocols and of genomic resources for some vegetable crops.

Family	Cultivated species	Estimated genome size (Mb)^a^	Regeneration and transformation^b^	Genomic resources^c^
Solanaceae	*Solanum lycopersicum*	900	++/+++	+++
	*Solanum melongena*	1130	+++	++
	*Capsicum annuum*	3300	-	++/+++
Brassicaceae	*Brassica rapa*	284	++	++
	*Brassica oleracea*	378	++	++
	*Raphanus sativus*	529	-	+
Cucurbitaceae	*Cucumis sativus*	350	++	+++
	*Cucumis melo*	375	++	+++
	*Citrullus lanatus*	425	++	++
	*Cucurbita pepo*	538	++	+
	*Cucurbita maxima*	450	++	-
Asteraceae	*Lactuca sativa*	2700	+++	+
	*Cichorium intybus*	1300–1400	++	+
	*Cynara cardunculus*	1084	-	++
Chenopodiaceae	*Spinacia oleracea*	1002	++	+
Apiaceae	*Daucus carota*	473	+++	++
	*Foeniculum vulgare*	4450	-/+	-
	*Apium graveolens*	3000	-/+	-
	*Petroselinum crispum*	2201	-	-
Leguminosae	*Phaseolus vulgaris*	587	+	+++
	*Vigna unguiculata*	620	+	-
	*Pisum sativum*	4300	+	-
	*Vicia faba*	13000	+	-
Amaryllidaceae	*Allium cepa*	16000	+/++	+
	*Allium sativum*	15901	+	-
	*Allium porrum*	28607	-	-
Asparagaceae	*Asparagus officinalis*	1308	-/*+*	+

*^a^*Solanum lycopersicum* (Tomato Genome Consortium, 2012); *S. melongena* (Hirakawa et al., 2014); *C. annuum* (Qin et al., 2014); *B. oleracea* (Liu et al., 2014); *B. rapa* (Wang X. et al., 2011); *R. sativus* (Kitashiba et al., 2014); *C. sativus* (Huang et al., 2009); *C. melo* (Garcia-Mas et al., 2012); *C. lanatus* (Guo et al., 2013); *C. pepo* (Plant DNA C-values database release 6.0, 2012 http://data.kew.org/cvalues/); *C. maxima* (Plant DNA C-values database release 6.0, 2012 http://data.kew.org/cvalues/); *L. sativa* (Truco et al., 2013); *C. intybus* (Gonthier et al., 2010); *C. cardunculus* (Scaglione et al., 2016); *S. oleracea* (Plant DNA C-values database release 6.0, 2012 http://data.kew.org/cvalues/); *D. carota* (Iorizzo et al., 2016); *F. vulgare* (Plant DNA C-values database release 6.0, 2012 http://data.kew.org/cvalues/); *A. graveolens* (Fu et al., 2013); *P. crispum* (Plant DNA C-values database release 6.0, 2012 http://data.kew.org/cvalues/); *P. vulgaris* (Schmutz et al., 2014); *V. unguiculata* (Timko et al., 2008); *P. sativum* (Macas et al., 2007); *V. faba* (Kaur et al., 2012); *A. cepa* (Finkers et al., 2015*)*; *A. sativum* (Sun et al., 2012); *A. porrum* (Plant DNA C-values database release 6.0, 2012 http://data.kew.org/cvalues/); *A. officinalis* (Li et al., 2014).*

*^b^“-” Protocols not available or available with low repeatability in a few genotypes⇒ “+++” various protocols (indirect or direct transformation methods in combination with different explant types, regeneration through DNSO or SE either from tissue explants or protoplasts) available for multiple genotypes.*

*^c^“-” Little information available ⇒ “+++” re-sequenced genomes from cultivated and wild species available in addition to the complete reference genome sequence; functional information available.*

## Genomic Resources

Next-Generation Sequencing technologies have determined a significant advancement in data generation. Indeed, large datasets are now being generated across various model and non-model plant species by sequencing whole genomes and/or by applying genome reduction strategies (i.e., RNA-sequencing, hybridization-based enrichment, restriction enzyme-based enrichment, etc.). The list of vegetable crops with publicly available complete or draft genome sequences is getting rich very quickly (Michael and VanBuren, 2015).

Within the Solanaceae family, complete genome sequences have been assembled, annotated, and published in different species, allowing the development of multiple publicly available resources^1^ (Fernandez-Pozo et al., 2015). The genomes of the cultivated tomato (Heinz 1706) and its wild ancestor (*S. pimpinellifolium*) were published in 2012 (Tomato Genome Consortium, 2012). The genome sequence of *S. pennellii*, a wild tomato species used as gene donor for the cultivated *S. lycopersicum* because of its extreme tolerance to abiotic stresses, was released in 2014 (Bolger et al., 2014). Its sequencing is enhancing the use of the already available introgression lines (ILs), in which genomic regions of *S. lycopersicum* are replaced with the corresponding segments of *S. pennellii*. Those publications allowed several re-sequencing projects to be developed with different scientific purposes (100 Tomato Genome Sequencing Consortium, 2014; Lin T. et al., 2014). Such recent efforts have led to the accumulation of a huge amount of valuable data on sequence diversity in the tomato clade that are of practical importance in the design of effective breeding strategies based on the generation of precise sequence changes in the target genome. In parallel, genome-wide association studies (GWAS) are revealing thousands of genetic variations associated with agronomically important traits (Ranc et al., 2012; Shirasawa et al., 2013; Ruggieri et al., 2014; Sauvage et al., 2014; Zhang J. et al., 2015), while much less numerous are the studies that rely on targeted enrichment methods for the discovery of genome-wide sequence variations from specific candidate *loci* (Terracciano et al., 2016).

Whole genome sequences of the hot (*C. annuum* cv. CM334) as well as of the cultivated pepper Zunla-1 (*C. annuum* L.) and its wild progenitor *Chiltepin* (*C. annuum* var. *glabriusculum*) have been released into the public domain together with re-sequencing data of cultivated, semi-wild/wild accessions (Kim et al., 2014; Qin et al., 2014). Genome resequencing of representative pepper accessions as well as single nucleotide polymorphism (SNP) discovery through genotyping by sequencing (Ahn et al., 2016; Taranto et al., 2016) have allowed genetic diversity in pepper to be unlocked. Nevertheless, the association between DNA variation and the observed phenotypic variability is still poor (Nimmakayala et al., 2016a).

A draft genome sequence of eggplant is also available (Hirakawa et al., 2014). An additional sequencing effort by an Italian consortium aiming at the generation of a gold-standard reference genome is ongoing (Barchi et al., 2016). Based on a fairly large number of accessions, a couple of GWAS have been published so far in eggplant allowing a series of novel marker/trait associations to be detected (Cericola et al., 2014; Portis et al., 2015).

Within the Brassicaceae, the sequencing of the *B. rapa* genome (Wang X. et al., 2011) has been followed by the release of the draft genome assembly of *B. oleracea* (Liu et al., 2014), a species characterized by a large morphological diversity and including different crops such as cauliflower, broccoli, cabbages, Brussels sprouts, kohlrabi, and kales. The investigation on the genetic variability in these species is still quite limited (Lin K. et al., 2014).

Within the Cucurbitaceae family, the complete genome of cucumber (*C. sativus* L.) and melon (*C. melo* L.) as well as the draft genome of watermelon (*C. lanatus*) and zucchini (*C. pepo*) are already available (Huang et al., 2009; Garcia-Mas et al., 2012; Guo et al., 2013)^2^. In case of *C. lanatus* the resequencing of 20 accessions has also been performed (Guo et al., 2013) providing a large source of haplotype data with great potential for next-generation breeding. In addition, medium to large SNP catalogs generated by genotyping by sequencing are available in watermelon (Nimmakayala et al., 2014) and melon (Nimmakayala et al., 2016b). The latter has been used to perform GWAS for fruit firmness. In the near future, it is also expected the publication of the pumpkin (*C. maxima* Duch.) genome sequence^3^.

The genome of the globe artichoke (*C. cardunculus* var. *scolymus*), a species belonging to the Asteraceae, was released in 2016 (Scaglione et al., 2016), while the first draft of the lettuce (*Lactuca sativa*) and chicory (*Cichorium intybus*) genomes has been announced^4^ (Galla et al., 2014). A transcriptome-based SNP discovery, through Illumina sequencing of 11 representative accessions of the three *C. cardunculus* taxa generated 195k variants (Scaglione et al., 2012). More recently, a re-sequencing approach, followed by a whole genome SNP mining strategy, as well as the identification of PAV (presence-absence variation), have been applied for globe artichoke (Acquadro et al., 2016).

A high quality chromosome-scale assembly of carrot (*Daucus carota* subsp. *carota* L.) genome is now available (Iorizzo et al., 2016) for the scientific community interested in the Apiaceae genetic improvement. Transcriptome-based analysis on the allelic diversity of wild and cultivated accession is also available (Iorizzo et al., 2013).

Common bean (*P. vulgaris* L.) genomes of two genotypes from Andean and Mesoamerican gene pools (Schmutz et al., 2014; Vlasova et al., 2016) have been published as part of the Leguminosae family. In addition, an International Consortium for Pea Genome Sequencing (PGS) has been formed to explore options for sequencing the pea genome that is particularly complex and large (∼4.3 Gb^5^). Several GWAS have been performed in common bean to explore broader genetic diversity in order to establish marker-trait associations for future application in breeding programs (Cichy et al., 2015; Kamfwa et al., 2015; Perseguini et al., 2016).

Knowledge on Amaryllidaceae and Asparagaceae genomes is scarce compared to Solanaceae and Cucurbitaceae. Genomic resources for garlic (*Allium sativum* L.), onion (*A. cepa* L.), and asparagus (*Asparagus officinalis* L.) are limited because of their large, extremely complex, repetitive, and often polyploid genomes and long generation times. Nevertheless, the Sequon – Onion Genome Sequencing project is underway^6^ with the main goal to generate a high quality sequence for the gene-rich regions of a doubled haploid (DH) onion line as a reference for the Amaryllidaceae family.

All these genome sequencing initiatives, usually paralleled by the generation of transcriptome-derived sequences in case of species of minor interest, have led to the development of clade-oriented databases dedicated to the genomics of specific crop families (Wang X. et al., 2011; Fernandez-Pozo et al., 2015)^7^. The genome size in various vegetables and the availability of genomic resources are summarized in **Table 1**.

## Genome Editing in Vegetable Crops

Current genome editing approaches rely on the induction of cuts in double-strand DNA (DSB, double-strand breaks), which are then “repaired” through two different processes: non-homologous end joining (NHEJ) or homology-directed repair (HDR; Cardi, 2016). In the former case, in the absence of foreign donor sequences, small changes (mostly frame-shift mutations due to insertions and deletions) are induced in the original sequence during the repair process, generally resulting in the loss of function of the target gene and a mutated phenotype. On the other hand, if appropriate DNA fragments homologous to the target sequence are also inserted into the cell, they can, using the precise HDR mechanism, replace (correct) some nucleotide sequences of the gene to be modified or add new genes or regulatory elements in a predetermined position of the genome. Genome editing strategies recently applied to vegetable crops are reported in **Table 2**. In all but one case, they have been based on the induction of targeted mutations through NHEJ repair of DSBs determined by either transcription activator-like effector nucleases (TALEN) or clustered regularly interspaced short palindromic repeats/CRISPR-associated 9 endonuclease system (CRISPR/Cas9).

**Table 2:** Genome editing approaches applied to vegetable crops.

Species	Trait	Gene	Genetic modification/double-strand breaks (DSB) repair mechanism^a^	Technology	DNA delivery	Tissue culture system	Reference
*Solanum lycopersicum* (tomato)	Plant development	*PROCERA (PRO)*	Indel mutations (gene knockout)/NHEJ	TALEN	*Agrobacterium tumefaciens* (binary vector)	Cotyledon explants	Lor et al., 2014
	Leaf development	*ARGONAUTE7 (SlAGO7)*	Indel mutations (gene knockout)/NHEJ	CRISPR/Cas9	*A. tumefaciens* (binary vector)	Cotyledon explants	Brooks et al., 2014
	Root development	*SHORT-ROOT (SHR)*	Indel mutations (gene knockout)/NHEJ	CRISPR/Cas9	*A. rhizogenes* (binary vector)	Cotyledon explants^b^	Ron et al., 2014
	Fruit ripening	*Ripening inhibitor (RIN)*	Indel mutations (gene knockout)/NHEJ	CRISPR/Cas9	*A. tumefaciens* (binary vector)	Cotyledon explants	Ito et al., 2015
	Anthocyanin biosynthesis	*Anthocyanin 1 (ANT1)*	Insertion of a novel promoter/HDR	TALEN, CRISPR/Cas9	*A. tumefaciens* (geminivirus replicons)	Cotyledon explants	Cermak et al., 2015
	Plant development	*Self-pruning 5G (sp5g), Self-pruning (sp)*	Indel mutations (gene knockout)/NHEJ	CRISPR/Cas9	*A. tumefaciens* (binary vector)	Cotyledon explants	Soyk et al., 2017
*Brassica oleracea* (broccoli^c^)	Plant development, fruit dehiscence	*Gibberellin 3-beta-dioxygenase 1 (BolC.GA4.a)*	Indel mutations (gene knockout)/NHEJ	CRISPR/Cas9	*A. tumefaciens* (binary vector)	Cotyledonary petiole explants	Lawrenson et al., 2015
*Lactuca sativa* (lettuce)	Plant development	*BRASSINOSTEROID INSENSITIVE 2 (BIN2)*	Indel mutations (gene knockout)/NHEJ	CRISPR/Cas9	Polyethylene glycol (PEG)^d^	Cotyledon protoplasts	Woo et al., 2015
*Cucumis sativus* (cucumber)	Virus resistance	*Eukaryotic translation initiation factor 4E (eIF4E)*	Indel mutations (gene knockout), SNPs/NHEJ	CRISPR/Cas9	*A. tumefaciens* (binary vector)	Cotyledon explants	Chandrasekaran et al., 2016

*^a^Double-Strand Breaks induced by sequence-specific nucleases were repaired either by non-homologous end joining (NHEJ) or homology-directed repair (HDR).*

*^b^Hairy roots were induced from explants after co-cultivation with *A. rhizogenes*.*

*^c^A doubled haploid genotype derived from a cross between Chinese broccoli (*B. oleracea* ssp. *alboglabra*) and broccoli (*B. oleracea* ssp. *italica*) was used.*

*^d^Preassembled complexes of purified Cas9 protein and guide RNA were transfected into plant protoplasts.*

In tomato, TALE nucleases under the control of an estrogen-inducible promoter have been used to knockout one DELLA gene (*PRO*) involved in negative regulation of gibberellic acid (GA) signaling. Consistently with the induction of frameshift mutations, loss of DELLA function and increased GA response, plants carrying two different mutant alleles (biallelic) or the same mutation in homozygous condition showed longer internodes than the wild type and lighter green leaves with smoother margins. Induced *pro* mutations were transmitted to the progenies according to Mendelian inheritance, while the transgene encoding the TALE nuclease segregated away from the TALEN-induced mutations and was not present in some of the plants showing the mutant phenotype (Lor et al., 2014). In the same species, a CRISPR/Cas9 construct has been designed to induce loss-of-function mutations in the *ARGONAUTE7* (*SlAGO7*) gene, which through the synthesis of short interfering RNAs causes post-transcriptional silencing of *AUXIN RESPONSE FACTOR* genes and regulates organ polarity (Brooks et al., 2014). Forty-eight percent of T_0_ transformed plants showed the expected phenotype with needle-like or wiry leaves. Only one regenerant, however, contained the deletion of the expected size after the contemporary use of two single guide RNAs (sgRNAs), the remaining being homozygous, biallelic or chimeric for small insertions and deletions. Induced mutations could be transmitted through the germline and the loss of the Cas9 transgene was shown in some progenies. A similar approach has been used in the same work to carry out functional studies in three homologs of the gene *Solyc11g064850* involved in multiple aspects of tomato reproductive development. CRISPR/Cas has also been used in functional studies aiming to understand, in a hairy root tissue culture system, the role of the tomato homolog of the transcription factor *SHORT-ROOT* (*SHR*), which in Arabidopsis is known to regulate the expression of another transcription factor (*SCARECROW, SCR*) and induce a short root phenotype (Ron et al., 2014). Three regions of the tomato *RIN* gene, encoding a MADS-box transcription factor regulating fruit ripening, have also been recently targeted for inducing sequence-specific mutations by NHEJ. A range of indel mutations have been detected already in T_0_ plants in homozygous, heterozygous, biallelic or chimeric condition. Homozygous plants for the desired mutation have been easily recovered in following generation and some T_1_ plants did not carry any T-DNA. Induced mutations provoked either the formation of a truncated RIN protein or no protein accumulation, affecting fruit ripening in a variety of modes. Differently from the conventional *rin* mutation, however, no other genes were affected (Ito et al., 2015). Recently, the CRISPR/Cas9 system has been utilized to inactivate two genes, *sp5g* (*self-pruning 5g*) and *sp* (*self-pruning*), involved in photoperiod response, flowering and control of determinate growth. Regardless of day length, “double-determinate CR-sp/sp5g plants” showed an earlier burst of flowering and earliness for fruit set when compared to indeterminate and determinate *sp*-classic controls (Soyk et al., 2017). Alternatively to frameshift mutations determined by imprecise NHEJ repair pathway, Cermak et al. (2015) pursued in tomato the precise insertion by homologous recombination (HR) of the strong constitutive promoter 35S upstream of endogenous *ANT1*, which encodes a Myb transcription factor and induces anthocyanin accumulation in purple tissues. Besides the 35S promoter and flanking recombination sequences, however, the donor template had to include a *nptII* gene for the selection of transformed cells. In order to increase template production in plant cells and gene targeting (GT) frequency, template and nuclease sequences (either TALEN or CRISPR/Cas9) have been cloned in place of endogenous viral genes encoding coat and movement proteins in a modified *Bean Yellow Dwarf Virus* (BeYDV) genome. As in previous reports in tomato, DNA delivery in plant cells has been accomplished by Agrobacterium-mediated transformation of cotyledon explants. By employing non-integrating geminivirus replicons GT frequency achieved by TALEN and CRISPR/Cas9 systems was comparable and about one order of magnitude higher than using non-replicating T-DNA vectors. Furthermore, no off-target mutations could be detected.

In *B. oleracea*, CRISPR/Cas9 has been used to induce indel mutations in two regions of the *BolC.GA4.a* gene, which similarly to the homolog *GA4* gene in Arabidopsis, is involved in gibberellin biosynthesis (Lawrenson et al., 2015). Regenerated plants showed a range of mutations in the target gene and two of them exhibited also the expected dwarf phenotype and alterations in pod valve margins. Some off-target mutations have been detected in another gene (*BolC.GA4.b*) which, compared with the *GA4.a* gene, showed two mismatches in target region 2 (no off-targeting, however, has been detected in target region 1 with four mismatches).

An alternative innovative approach for delivering editing reagents in plant cells has been recently reported in a number of species, including lettuce (Woo et al., 2015). In the latter, the homolog of the Arabidopsis BRASSINOSTEROID INSENSITIVE 2 (BIN2) gene, encoding a negative regulator in the brassinosteroid (BR) signaling pathway, has been knocked-out after transfecting PEG-treated protoplasts with a mixture of Cas9 and a sgRNA targeting the third exon of the gene. Overall mutation frequency in protoplast-derived calli was ∼46%: 5.7 and 40% of calli contained monoallelic and biallelic mutations at the target site, respectively. No off-target mutations were detected and plants regenerated *via* organogenesis from mutant calli transmitted the mutations to the progeny.

Finally, the virus resistance of cucumber plants has been investigated after mutating the Eukaryotic translation initiation factor 4E (*eIF4E*) gene in two sites: in the first case, the gene was completely knocked-down, whereas, in the second, translation of two-thirds of the protein product was still possible (Chandrasekaran et al., 2016). Chimeric T_0_ plants containing indel and SNP mutations were regenerated and T_3_ progenies were submitted to virus tests. Non-transgenic homozygous mutant plants showed either immunity or resistance to *Cucumber vein yellowing virus* (CVYV), *Zucchini yellow mosaic virus* (ZYMV), and *Papaya ring spot mosaic virus-W* (PRSV-W), although resistance breaking was observed in some cases. The same plants had no resistance to *Cucumber mosaic virus* (CMV) and *Cucumber green mottle mosaic virus* (CGMMV). After specific analyses, no off-target activity could be detected.

## Conclusion and Perspectives

Vegetables include a broad number of species with different reproductive features and genetic structures, which largely affect the access to and the exploitation of genetic variability as well as the efficiency of breeding efforts. In the past decades, genetic improvement programs relied on the selection of superior families or plants from intra-specific or inter-specific cross-derived populations, involving the combination of favorable traits from different genetic backgrounds. The low efficiency of phenotypic selection, especially for quantitatively inherited agronomic traits, has been overcome by the introduction of molecular marker technologies that have improved the efficiency of selection, allowing the detection of specific regions and/or genes to introgress *via* MAB (marker assisted breeding) programs. These approaches, however, are not free from limitations due to the large number of crosses and derived plants to manage. Artificial mutagenesis, developed in the ‘60, allowed to increase the range of variability available for direct or indirect use, with beneficial effects particularly in vegetatively propagated crops, but the necessity to recover the desirable trait in very large populations composed of plants carrying also many undesirable mutations limits its use.

Priorities in the development of novel vegetable cultivars include the improvement of quality traits, the introduction of resistance to a broad range of pests and pathogens, and tolerance to abiotic stresses such as drought, salinity, and low/high temperature. Most of these resistance/tolerance traits need often to be transferred from wild germplasm, taking many generations to remove the deleterious genes that go along with the desirable traits due to linkage drag (Bai and Lindhout, 2007). In some cases, genes are in secondary or tertiary gene pools requiring tissue culture and/or cytogenetic manipulations in order to obtain fertile hybrids and offsprings. Furthermore, the polygenic nature of many agricultural and biochemical characters warrants the development of extensive trial populations and appropriate experimental designs for the identification of the underlying genes.

**FIGURE 2:**
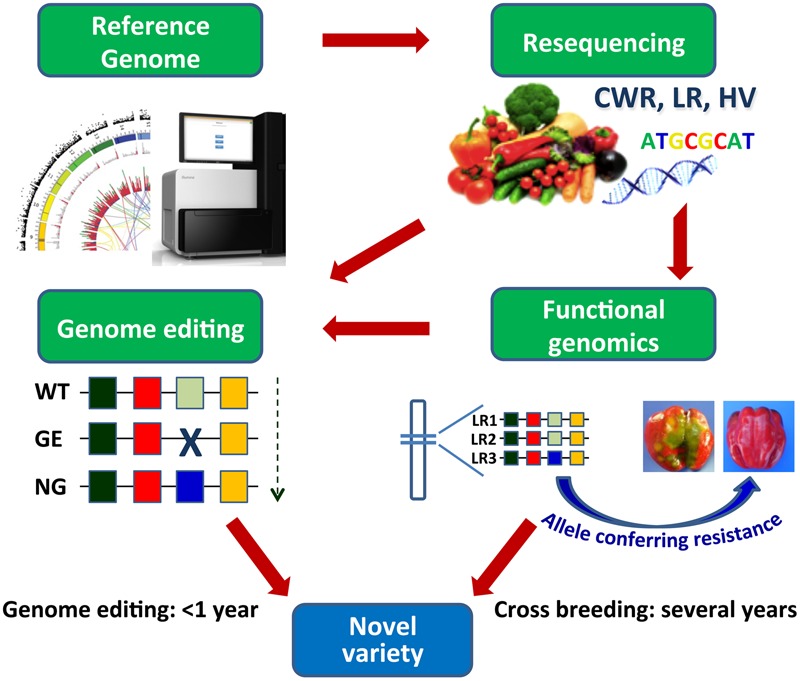
**Development and exploitation of genomic resources by cross breeding and by genome editing.** Resequencing of CWR, LR, and heirloom varieties (HV) in vegetables and subsequent identification of the allelic variation associated to relevant traits allow to introduce precise genome modifications accelerating the breeding of novel varieties. WT, wild type; GE, genome editing process; NG, new “genome edited” genotype; LR1, 2, 3, landraces. Alleles are indicated by colored boxes.

Genome editing approaches, aiming to either functional studies or genetic improvement, help face some of the above-mentioned issues and allow to overcome some limitations of classical breeding (Abdallah et al., 2015; Nogue et al., 2016; Rani et al., 2016) (**Figure 2**). The possibility to easily recover in regenerated plants or first generation progenies homozygous/biallelic mutations induced by targeted mutagenesis or other editing approaches will ease the production of novel sets of parental lines to be used in F_1_ hybrid production. An additional advantage consists in the possibility to produce modified plants eventually non-transgenic, by expressing editing reagents transiently or inducing sorting-out of transgenic sequences by segregation or by the use of recombinases (Wang Y. et al., 2011; Mahfouz et al., 2016). The possibility to use genome editing tools either to achieve the crop ideotype by modifying major genes underlying key vegetative and reproductive traits, or induce the *de novo* domestication of wild relatives by manipulating monogenic yield-related traits, has been recently exemplified in tomato (Zsögön et al., 2017).

In the last 20 years, several efforts have been made to achieve precise targeted mutagenesis and gene insertion in higher plants, but only the development of site directed nucleases, especially the CRISPR/Cas system, has allowed a more widespread use of such approaches (Cardi and Stewart, 2016). Nevertheless, compared to cereals and other major crops (e.g., potato or oilseed rape), their application is still limited to few vegetable crops and traits. Besides regulatory and patenting issues, that will be hopefully sorted out in a near future (Egelie et al., 2016; Schinkel and Schillberg, 2016; Sprink et al., 2016), major limiting factors for a more common application of genome editing to cultivated vegetable genotypes include the availability of genomic information as well as of efficient protocols for transformation and regeneration. Furthermore, genome editing components have to be efficiently delivered into plant cells and, in case of modifications *via* HDR, frequency of HR optimized (Voytas, 2013; Altpeter et al., 2016; Steinert et al., 2016).

Whole genome sequences of many vegetable crops are already available, while allele mining efforts, based on whole genome resequencing and/or targeted resequencing of a fairly large number of accessions, are underway to search out valuable allelic variants in landraces and crop wild relatives. These efforts are generally paralleled by GWAS that are revealing a series of novel marker/trait associations. All such sequence-based knowledge is an essential prerequisite to transfer technologically advanced methods to vegetable species, allowing overcoming the barriers to a more widespread use of new technologies. Indeed, the possibility to use genome editing approaches to selectively knockout target genes or induce specific mutations is also effective to understand gene functions.

Although efficient ways to deliver editing reagents in plant cells must be still achieved in most crops (Ledford, 2016), it can be envisaged that in vegetables with substantial information on gene sequence and function as well as good regeneration/transformation potential (e.g., tomato, eggplant, lettuce), a range of editing approaches including gene knockout, gene editing or gene replacement/stacking can be applied soon to induce recessive or dominant novel traits. On the other hand, in cases where tissue culture and/or genomic resources are available but still not optimal, applications of genome editing will be likely limited to the introduction of (recessive) frameshift mutations in target genes, for instance those encoding negative regulators or factors necessary for essential pathogen functions [susceptibility (S) genes] (van Schie and Takken, 2014; Sun et al., 2016). In addition, provided that information on regulatory sequences are available, modified CRISPR/Cas9 complexes can be used to mediate the transcriptional activation/repression of the expression of endogenous genes (Piatek and Mahfouz, 2016). In case transformation and/or regeneration protocols are lacking in cultivated genotypes of interest, genome editing approaches can be applied to selected genotypes with good transformation/regeneration ability in order to introduce relevant mutations in the primary gene pool and subsequently transfer them by conventional means. Nevertheless, in some important vegetables (e.g., artichoke, pepper, legumes) it seems necessary to focus on the development of repeatable regeneration/transformation protocols.

The development of such protocols has so far largely been the result of empirical experimentation regarding gene delivery and culture parameters. As previously reported, however, the genetic control of regeneration and transformation ability has been established in various species and the transfer of responsible genes achieved in some instances. More recently, the function of a number of genes involved in genetic and epigenetic mechanisms underpinning various steps of either DNSO or SE, and their possible manipulation for improving response of recalcitrant genotypes, have been described in several reviews (Duclercq et al., 2011; Pulianmackal et al., 2014; Xu and Huang, 2014; Ikeuchi et al., 2016). Engineering the production of specific plant and bacterial proteins could also help enhance Agrobacterium-mediated transformation (Gelvin, 2003; Altpeter et al., 2016). In order to increase feasibility of massive screening of edited products, an automated platform has been recently developed for transformation and genome editing using plant protoplasts (Dlugosz et al., 2016). Alternatively, transformation protocols not relying on tissue culture and *in vitro* regeneration should be developed in recalcitrant genotypes (Altpeter et al., 2016), but although they have been reported in several crops, as described above, their reliability and general applicability is questioned (Finer, 2010).

As far as future applications are concerned, virus resistance represents a major concern in many vegetable crops. Besides recessive resistance achieved by manipulating host genes coding for factors required by invading viruses, as previously discussed for cucumber (Chandrasekaran et al., 2016), acquisition of dominant resistance has been recently reported by expressing in *Nicotiana benthamiana* sgRNAs directed toward various ssDNA Geminivirus, that replicate in plant cells through an intermediate dsDNA stage and are known to affect a large number of vegetables (Zaidi et al., 2016). Results obtained with *tomato yellow leaf curl virus* (TYLCV) and BeYDV are particularly interesting considering their possible transfer in vegetable host species (Ali et al., 2015; Baltes et al., 2015).

To date, genome editing has been mainly focused on the control of single variants underlying qualitative traits. Quantitative variation is instead mediated by several nucleotides (QTN, quantitative trait nucleotides) with large and small effects on the phenotype. Editing quantitative traits is feasible once the availability of datasets of sequences and phenotypes will enable to discover large numbers of QTNs (Jenko et al., 2015). A possible future achievement could be to perform a small number of edits on few QTNs with major effects. A further constraint in vegetable breeding is the manipulation of the reproductive system (e.g., apomixis and self-incompatibility). Such traits are under the control of several candidate genes (Hand and Koltunow, 2014; Yamamoto and Nishio, 2014) and genome editing methods could facilitate the identification of their roles, enhancing the possibility to fix desirable genotypes and accelerate the breeding rate.

## Author Contributions

TC, NDA, and PT conceived and wrote the manuscript. All authors read and approved the final manuscript.

## Conflict of Interest Statement

The authors declare that the research was conducted in the absence of any commercial or financial relationships that could be construed as a potential conflict of interest.

## Footnotes

1 https://solgenomics.net/

2 https://cucurbigene.upv.es/genome-v3.2/

3 http://www.icugi.org/cgi-bin/ICuGI/index.cgi

4 https://lgr.genomecenter.ucdavis.edu/

5 https://www.france-genomique.org/spip/spip.php?article141&lang=fr

6 http://www.oniongenome.net/

7 http://www.icugi.org/cgi-bin/ICuGI/index.cgi

## References

[B1] 100 Tomato Genome Sequencing Consortium, Aflitos S. Schijlen E. De Jong H. De Ridder D. Smit S. (2014). Exploring genetic variation in the tomato (*Solanum* section Lycopersicon) clade by whole-genome sequencing. *Plant J.* 80 136–148. 10.1111/tpj.12616 25039268

[B2] Abdallah N. A. Prakash C. S. Mchughen A. G. (2015). Genome editing for crop improvement: challenges and opportunities. *GM Crops Food* 6 183–205. 10.1080/21645698.2015.1129937 26930114 PMC5033222

[B3] Acquadro A. Barchi L. Portis E. Mangino G. Valentino D. Mauromicale G. (2016). “Whole genome resequencing in *Cynara cardunculus*: detection of intra-specific variability,” in *Proceedings of the 20th EUCARPIA General Congress: Plant Breeding: the Art of Bringing Science to Life* Zürich 334.

[B4] Ahn Y.-K. Karna S. Jun T.-H. Yang E.-Y. Lee H.-E. Kim J.-H. (2016). Complete genome sequencing and analysis of *Capsicum annuum* varieties. *Mol. Breed.* 36:140 10.1007/s11032-016-0557-9

[B5] Ahn Y. K. Yoon M. K. Jeon J. S. (2013). Development of an efficient agrobacterium-mediated transformation system and production of herbicide-resistant transgenic plants in garlic (*Allium sativum* L.). *Mol. Cells* 36 158–162. 10.1007/s10059-013-0142-6 23832764 PMC3887948

[B6] Ali Z. Abulfaraj A. Idris A. Ali S. Tashkandi M. Mahfouz M. M. (2015). CRISPR/Cas9-mediated viral interference in plants. *Genome Biol.* 16:238 10.1186/s13059-015-0799-6 PMC4641396 26556628

[B7] Altpeter F. Springer N. M. Bartley L. E. Blechl A. E. Brutnell T. P. Citovsky V. (2016). Advancing crop transformation in the era of genome editing. *Plant Cell* 28 1510–1520. 10.1105/tpc.16.00196 27335450 PMC4981132

[B8] Anderson P. K. Cunningham A. A. Patel N. G. Morales F. J. Epstein P. R. Daszak P. (2004). Emerging infectious diseases of plants: pathogen pollution, climate change and agrotechnology drivers. *Trends Ecol. Evol.* 19 535–544. 10.1016/j.tree.2004.07.021 16701319

[B9] Anzidei M. Bennici A. Schiff S. Tani C. Mori B. (2000). Organogenesis and somatic embryogenesis in *Foeniculum vulgare*: histological observations of developing embryogenic callus. *Plant Cell Tissue Organ Cult.* 61 69–79. 10.1023/A:1006454702620

[B10] Aragão F. J. L. Campos F. A. P. (2007). “Common bean and cowpea,” in *Transgenic Crops IV* eds Pua E.-C. Davey M. R. (Heidelberg: Springer Berlin Heidelberg) 263–276. 10.1007/978-3-540-36752-9_14

[B11] Aswath C. R. Mo S. Y. Kim D. H. Park S. W. (2006). *Agrobacterium* and biolistic transformation of onion using non-antibiotic selection marker phosphomannose isomerase. *Plant Cell Rep.* 25 92–99. 10.1007/s00299-005-0022-4 16211408

[B12] Bai Y. Lindhout P. (2007). Domestication and breeding of tomatoes: what have we gained and what can we gain in the future? *Ann. Bot.* 100 1085–1094. 10.1093/aob/mcm150 17717024 PMC2759208

[B13] Bakshi S. Saha B. Roy N. K. Mishra S. Panda S. K. Sahoo L. (2012). Successful recovery of transgenic cowpea (*Vigna unguiculata*) using the 6-phosphomannose isomerase gene as the selectable marker. *Plant Cell Rep.* 31 1093–1103. 10.1007/s00299-012-1230-3 22327900

[B14] Bakshi S. Sahoo L. (2013). How relevant is recalcitrance for the recovery of transgenic cowpea: implications of selection strategies. *J. Plant Growth Regul.* 32 148–158. 10.1007/s00344-012-9284-6

[B15] Baltes N. J. Hummel A. W. Konecna E. Cegan R. Bruns A. N. Bisaro D. M. (2015). Conferring resistance to geminiviruses with the CRISPR–Cas prokaryotic immune system. *Nat. Plants* 1:15145 10.1038/nplants.2015.145 PMC8612103 34824864

[B16] Baranski R. (2008). Genetic transformation of carrot (*Daucus carota*) and other Apiaceae species. *Transgenic Plant J.* 2 18–38.

[B17] Barchi L. Delledonne M. Lanteri S. Dal Molin A. Minio A. Ferrarini A. (2016). “A high quality eggplant genome sequence: a new tool for the analysis of Solanaceae family evolution and for the molecular deciphering of complex traits,” in *Proceedings of the 20th EUCARPIA General Congress* Zurich.

[B18] Barraza A. Cabrera-Ponce J. L. Gamboa-Becerra R. Luna-Martinez F. Winkler R. Alvarez-Venegas R. (2015). The *Phaseolus vulgaris* PvTRX1h gene regulates plant hormone biosynthesis in embryogenic callus from common bean. *Front. Plant Sci.* 6:577 10.3389/fpls.2015.00577 PMC4516878 26284093

[B19] Baskaran P. Soós V. Balázs E. Van Staden J. (2016). Shoot apical meristem injection: a novel and efficient method to obtain transformed cucumber plants. *S. Afr. J. Bot.* 103 210–215. 10.1016/j.sajb.2015.09.006

[B20] Bezirganoglu I. Hwang S. Y. Shaw J. F. Fang T. J. (2014). Efficient production of transgenic melon via *Agrobacterium*-mediated transformation. *Genet. Mol. Res.* 13 3218–3227. 10.4238/2014.April.25.7 24841654

[B21] Bolger A. Scossa F. Bolger M. E. Lanz C. Maumus F. Tohge T. (2014). The genome of the stress-tolerant wild tomato species *Solanum pennellii*. *Nat. Genet.* 46 1034–1038. 10.1038/ng.3046 25064008 PMC7036041

[B22] Brooks C. Nekrasov V. Lippman Z. B. Van Eck J. (2014). Efficient gene editing in tomato in the first generation using the clust ered regularly interspaced short palindromic repeats/CRISPR-associated9 system. *Plant Physiol.* 166 1292–1297. 10.1104/pp.114.247577 25225186 PMC4226363

[B23] Cardi T. (2016). Cisgenesis and genome editing: combining concepts and efforts for a smarter use of genetic resources in crop breeding. *Plant Breed.* 135 139–147. 10.1111/pbr.12345

[B24] Cardi T. Stewart C. N. Jr (2016). Progress of targeted genome modification approaches in higher plants. *Plant Cell Rep.* 35 1401–1416. 10.1007/s00299-016-1975-1 27025856

[B25] Cericola F. Portis E. Lanteri S. Toppino L. Barchi L. Acciarri N. (2014). Linkage disequilibrium and genome-wide association analysis for anthocyanin pigmentation and fruit color in eggplant. *BMC Genomics* 15:896 10.1186/1471-2164-15-896 PMC4210512 25311640

[B26] Cermak T. Baltes N. J. Cegan R. Zhang Y. Voytas D. F. (2015). High-frequency, precise modification of the tomato genome. *Genome Biol.* 16 232 10.1186/s13059-015-0796-9 PMC4635538 26541286

[B27] Chandrasekaran J. Brumin M. Wolf D. Leibman D. Klap C. Pearlsman M. (2016). Development of broad virus resistance in non-transgenic cucumber using CRISPR/Cas9 technology. *Mol. Plant Pathol.* 17 1140–1153. 10.1111/mpp.12375 26808139 PMC6638350

[B28] Chen W. Chiu C. Liu H. Lee T. Cheng J. Lin C. (1998). Gene transfer via pollen-tube pathway for anti-fusarium wilt in watermelon. *Biochem. Mol. Biol. Int.* 46 1201–1209. 10.1080/15216549800204762 9891853

[B29] Chin D. P. Bao J. H. Mii M. (2009). Transgenic spinach plants produced by *Agrobacterium*-mediated method based on the low temperature-dependent high plant regeneration ability of leaf explants. *Plant Biotechnol.* 26 243–248. 10.5511/plantbiotechnology.26.243

[B30] Chowrira G. M. Akella V. Fuerst P. E. Lurquin P. F. (1996). Transgenic grain legumes obtained byin planta electroporation-mediated gene transfer. *Mol. Biotechnol.* 5 85–96. 10.1007/BF02789058 8734422

[B31] Cichy K. A. Wiesinger J. A. Mendoza F. A. (2015). Genetic diversity and genome-wide association analysis of cooking time in dry bean (*Phaseolus vulgaris* L.). *Theor. Appl. Genet.* 128 1555–1567. 10.1007/s00122-015-2531-z 26003191

[B32] Citadin C. T. Ibrahim A. B. Aragão F. J. L. (2011). Genetic engineering in Cowpea (*Vigna unguiculata*): history, status and prospects. *GM Crops* 2 144–149. 10.4161/gmcr.2.3.18069 22179190

[B33] Curtis I. S. Nam H. G. (2001). Transgenic radish (*Raphanus sativus* L. longipinnatus Bailey) by floral-dip method – plant development and surfactant are important in optimizing transformation efficiency. *Transgenic Res.* 10 363–371. 10.1023/A:1016600517293 11592715

[B34] Davey M. R. Anthony P. Van Hooff P. Power J. B. Lowe K. C. (2007). “Lettuce,” in *Biotechnology in Agriculture and Forestry. Transgenic Crops IV* eds Pua E. C. Davey M. R. (Heidelberg: Springer-Verlag) 221–249. 10.1007/978-3-540-36752-9_12

[B35] Derks F. H. M. (1992). *Organelle Transfer by Protoplast Fusion in Solanaceae.* Ph.D. thesis, Free University of Amsterdam Amsterdam.

[B36] Desjardins Y. (2014). “Fruit and vegetables and health: an overview,” in *Horticulture: Plants for People and Places* eds Dixon G. R. Aldous D. E. (Dordrecht: Springer Science+Business Media) 965–1000. 10.1007/978-94-017-8560-0_2

[B37] Dewir Y. H. Murthy H. N. Ammar M. H. Alghamdi S. S. Al-Suhaibani N. A. Alsadon A. A. (2016). In vitro rooting of leguminous plants: difficulties, alternatives, and strategies for improvement. *Hortic. Environ. Biotechnol.* 57 311–322. 10.1007/s13580-016-0060-6

[B38] Dlugosz E. M. Lenaghan S. C. Stewart C. N. Jr. (2016). A robotic platform for high-throughput protoplast isolation and transformation. *J. Vis. Exp.* 2016:e54300 10.3791/54300 PMC5092064 27768035

[B39] Duclercq J. Sangwan-Norreel B. Catterou M. Sangwan R. S. (2011). De novo shoot organogenesis: from art to science. *Trends Plant Sci.* 16 597–606. 10.1016/j.tplants.2011.08.004 21907610

[B40] Eady C. Davis S. Catanach A. Kenel F. Hunger S. (2005). *Agrobacterium tumefaciens*-mediated transformation of leek (*Allium porrum*) and garlic (*Allium sativum*). *Plant Cell Rep.* 24 209–215. 10.1007/s00299-005-0926-z 15789208

[B41] Eady C. C. Weld R. J. Lister C. E. (2000). *Agrobacterium tumefaciens*-mediated transformation and transgenic-plant regeneration of onion (*Allium cepa* L.). *Plant Cell Rep.* 19 376–381. 10.1007/s002990050743 30754790

[B42] Egelie K. J. Graff G. D. Strand S. P. Johansen B. (2016). The emerging patent landscape of CRISPR-Cas gene editing technology. *Nat. Biotechnol.* 34 1025–1031. 10.1038/nbt.3692 27727218

[B43] Fernandez-Pozo N. Menda N. Edwards J. D. Saha S. Tecle I. Y. Strickler S. R. (2015). The Sol Genomics Network (SGN)–from genotype to phenotype to breeding. *Nucleic Acids Res.* 43 D1036–D1041. 10.1093/nar/gku1195 25428362 PMC4383978

[B44] Finer J. J. (2010). “Plant nuclear transformation,” in *Genetic Modification of Plants* 1st Edn eds Kempken F. Jung C. (Heidelberg: Springer-Verlag) 499–550.

[B45] Finkers R. Van Workum W. Van Kaauwen M. Huits H. Jungerius A. Vosman B. (2015). “A de novo assembly for the 16GB *Allium cepa* genome, tears of joy,” in *Proceedings of the Largest Ag-Genomics Meeting in the World: Plant & Animal Genome XXIII* San Diego, CA.

[B46] Fu N. Wang Q. Shen H. L. (2013). De novo assembly, gene annotation and marker development using Illumina paired-end transcriptome sequences in celery (*Apium graveolens* L.). *PLoS ONE* 8:e57686 10.1371/journal.pone.0057686 PMC3585167 23469050

[B47] Galla G. Chedina A. Tiozzo S. Barcaccia G. (2014). “Towards a first high-quality genome draft for leaf chicory, radicchio (*Cichorium intybus* L.),” in *Proceedings of the 58th Italian Society of Agricultural Genetics Annual Congress* Alghero.

[B48] Gantait S. Mandal N. Das P. K. (2010). An Overview on in vitro culture of genus allium. *Am. J. Plant Physiol.* 5 325–337. 10.3923/ajpp.2010.325.337

[B49] Garcia-Mas J. Benjak A. Sanseverino W. Bourgeois M. Mir G. Gonzalez V. M. (2012). The genome of melon (*Cucumis melo* L.). *Proc. Natl. Acad. Sci. U.S.A.* 109 11872–11877. 10.1073/pnas.1205415109 22753475 PMC3406823

[B50] Gatti I. Guindón F. Bermejo C. Espósito A. Cointry E. (2016). In vitro tissue culture in breeding programs of leguminous pulses: use and current status. *Plant Cell Tissue Organ Cult.* 127 543–559. 10.1007/s11240-016-1082-6

[B51] Geekiyanage S. Takase T. Watanabe S. Fukai S. Kiyosue T. (2006). The combined effect of photoperiod, light intensity and GA_3_ on adventitious shoot regeneration from cotyledons of spinach (*Spinacia oleracea* L.). *Plant Biotechnol.* 23 431–435. 10.5511/plantbiotechnology.23.431

[B52] Gelvin S. B. (2003). Improving plant genetic engineering by manipulating the host. *Trends Biotechnol.* 21 95–98. 10.1016/S0167-7799(03)00005-2 12628361

[B53] Gómez-Montes E. O. Oliver-Salvador C. Durán-Figueroa N. Badillo-Corona J. A. Salas C. E. (2015). Optimization of direct shoot regeneration using cotyledonary explants and true leaves from lettuce cv. Romaine (*Lactuca sativa* L.) by surface response methodology. *Plant Growth Regul.* 77 327–334. 10.1007/s10725-015-0067-5

[B54] Gonthier L. Bellec A. Blassiau C. Prat E. Helmstetter N. Rambaud C. (2010). Construction and characterization of two BAC libraries representing a deep-coverage of the genome of chicory (*Cichorium intybus* L., Asteraceae). *BMC Res. Notes* 3:225 10.1186/1756-0500-3-225 PMC2933585 20701751

[B55] Guo S. Zhang J. Sun H. Salse J. Lucas W. J. Zhang H. (2013). The draft genome of watermelon (*Citrullus lanatus*) and resequencing of 20 diverse accessions. *Nat. Genet.* 45 51–58. 10.1038/ng.2470 23179023

[B56] Hand M. L. Koltunow A. M. (2014). The genetic control of apomixis: asexual seed formation. *Genetics* 197 441–450. 10.1534/genetics.114.163105 24939990 PMC4063905

[B57] Heidmann I. De Lange B. Lambalk J. Angenent G. C. Boutilier K. (2011). Efficient sweet pepper transformation mediated by the BABY BOOM transcription factor. *Plant Cell Rep.* 30 1107–1115. 10.1007/s00299-011-1018-x 21305301 PMC3092944

[B58] Hirakawa H. Shirasawa K. Miyatake K. Nunome T. Negoro S. Ohyama A. (2014). Draft genome sequence of eggplant (*Solanum melongena* L.): the representative *solanum* species indigenous to the old world. *DNA Res.* 21 649–660. 10.1093/dnares/dsu027 25233906 PMC4263298

[B59] Hnatuszko-Konka K. Kowalczyk T. Gerszberg A. Wiktorek-Smagur A. Kononowicz A. K. (2014). *Phaseolus vulgaris* — Recalcitrant potential. *Biotechnol. Adv.* 32 1205–1215. 10.1016/j.biotechadv.2014.06.001 24953179

[B60] Huang S. Li R. Zhang Z. Li L. Gu X. Fan W. (2009). The genome of the cucumber, *Cucumis sativus* L. *Nat. Genet.* 41 1275–1281. 10.1038/ng.475 19881527

[B61] Ikeuchi M. Ogawa Y. Iwase A. Sugimoto K. (2016). Plant regeneration: cellular origins and molecular mechanisms. *Development* 143 1442–1451. 10.1242/dev.134668 27143753

[B62] Ilori C. O. Pellegrineschi A. (2011). Transgene expression in cowpea (*Vigna unguiculata* (L.) Walp.) through *Agrobacterium* transformation of pollen in flower buds. *Afr. J. Biotechnol.* 10 11821–11828.

[B63] Iorizzo M. Ellison S. Senalik D. Zeng P. Satapoomin P. Huang J. (2016). A high-quality carrot genome assembly provides new insights into carotenoid accumulation and asterid genome evolution. *Nat. Genet.* 48 657–666. 10.1038/ng.3565 27158781

[B64] Iorizzo M. Senalik D. A. Ellison S. L. Grzebelus D. Cavagnaro P. F. Allender C. (2013). Genetic structure and domestication of carrot (*Daucus carota* subsp. sativus) (Apiaceae). *Am. J. Bot.* 100 930–938. 10.3732/ajb.1300055 23594914

[B65] Ito Y. Nishizawa-Yokoi A. Endo M. Mikami M. Toki S. (2015). CRISPR/Cas9-mediated mutagenesis of the RIN locus that regulates tomato fruit ripening. *Biochem. Biophys. Res. Commun.* 467 76–82. 10.1016/j.bbrc.2015.09.117 26408904

[B66] Jakhar M. L. Choudhary M. R. (2012). Regeneration of in vitro plantlets through organogenesis in fennel (*Foeniculum Vulgare* Mill.). *J. Plant Sci. Res.* 28 203–209.

[B67] Jenko J. Gorjanc G. Cleveland M. A. Varshney R. K. Whitelaw C. B. Woolliams J. A. (2015). Potential of promotion of alleles by genome editing to improve quantitative traits in livestock breeding programs. *Genet. Sel. Evol.* 47 55 10.1186/s12711-015-0135-3 PMC4487592 26133579

[B68] Kamfwa K. Cichy K. A. Kelly J. D. (2015). Genome-wide association analysis of symbiotic nitrogen fixation in common bean. *Theor. Appl. Genet.* 128 1999–2017. 10.1007/s00122-015-2562-5 26133733

[B69] Kaur S. Pembleton L. W. Cogan N. O. Savin K. W. Leonforte T. Paull J. (2012). Transcriptome sequencing of field pea and faba bean for discovery and validation of SSR genetic markers. *BMC Genomics* 13:104 10.1186/1471-2164-13-104 PMC3352077 22433453

[B70] Kenel F. Eady C. Brinch S. (2010). Efficient *Agrobacterium tumefaciens*-mediated transformation and regeneration of garlic (*Allium sativum*) immature leaf tissue. *Plant Cell Rep.* 29 223–230. 10.1007/s00299-009-0814-z 20099065

[B71] Kim S. Park M. Yeom S. I. Kim Y. M. Lee J. M. Lee H. A. (2014). Genome sequence of the hot pepper provides insights into the evolution of pungency in *Capsicum* species. *Nat. Genet.* 46 270–278. 10.1038/ng.2877 24441736

[B72] Kitashiba H. Li F. Hirakawa H. Kawanabe T. Zou Z. Hasegawa Y. (2014). Draft sequences of the radish (*Raphanus sativus* L.) *genome*. *DNA Res.* 21 481–490. 10.1093/dnares/dsu014 24848699 PMC4195494

[B73] Klenotičová H. Smykalová I. Švábová L. Griga M. (2013). Resolving browning during the establishment of explant cultures in Vicia faba L. for genetic transformation. *Acta Univ. Agric. Silvic. Mendel. Brun.* 61 1279–1288. 10.11118/actaun201361051279

[B74] Klocke E. Nothnagel T. Schumann G. (2010). “Vegetables,” in *Genetic Modification of Plants* 1st Edn eds Kempken F. Jung C. (Heidelberg: Springer-Verlag).

[B75] Koornneef M. Bade J. Hanhart C. Horsman K. Schel J. Soppe W. (1993). Characterization and mapping of a gene controlling shoot regeneration in tomato. *Plant J.* 3 131–141. 10.1111/j.1365-313X.1993.tb00016.x

[B76] Kothari S. L. Joshi A. Kachhwaha S. Ochoa-Alejo N. (2010). Chilli peppers–a review on tissue culture and transgenesis. *Biotechnol. Adv.* 28 35–48. 10.1016/j.biotechadv.2009.08.005 19744550

[B77] Kumar P. Srivastava D. K. (2016). Biotechnological advancement in genetic improvement of broccoli (*Brassica oleracea* L. var. italica), an important vegetable crop. *Biotechnol. Lett.* 38 1049–1063. 10.1007/s10529-016-2080-9 26971329

[B78] Kurtz S. M. Lineberger R. D. (1983). Genotypic differences in morphogenic capacity of cultured leaf explants of tomato [*Lycopersicon esculentum*]. *J. Am. Soc. Hortic. Sci.* 108 710–714.

[B79] Lawrenson T. Shorinola O. Stacey N. Li C. Ostergaard L. Patron N. (2015). Induction of targeted, heritable mutations in barley and *Brassica oleracea* using RNA-guided Cas9 nuclease. *Genome Biol.* 16 258 10.1186/s13059-015-0826-7 PMC4663725 26616834

[B80] Ledford H. (2016). A better way to hack plant DNA. *Nature* 539 16–17. 10.1038/539016a 27808215

[B81] Li S. F. Gao W. J. Zhao X. P. Dong T. Y. Deng C. L. Lu L. D. (2014). Analysis of transposable elements in the genome of *Asparagus officinalis* from high coverage sequence data. *PLoS ONE* 9:e97189 10.1371/journal.pone.0097189 PMC4014616 24810432

[B82] Lin K. Zhang N. Severing E. I. Nijveen H. Cheng F. Visser R. G. (2014). Beyond genomic variation–comparison and functional annotation of three *Brassica rapa* genomes: a turnip, a rapid cycling and a Chinese cabbage. *BMC Genomics* 15:250 10.1186/1471-2164-15-250 PMC4230417 24684742

[B83] Lin T. Zhu G. Zhang J. Xu X. Yu Q. Zheng Z. (2014). Genomic analyses provide insights into the history of tomato breeding. *Nat. Genet.* 46 1220–1226. 10.1038/ng.3117 25305757

[B84] Liu L. Gu Q. Ijaz R. Zhang J. Ye Z. (2016). Generation of transgenic watermelon resistance to *Cucumber mosaic virus* facilitated by an effective *Agrobacterium*-mediated transformation method. *Sci. Hortic.* 205 32–38. 10.1016/j.scienta.2016.04.013

[B85] Liu S. Liu Y. Yang X. Tong C. Edwards D. Parkin I. A. (2014). The *Brassica oleracea* genome reveals the asymmetrical evolution of polyploid genomes. *Nat. Commun.* 5:3930 10.1038/ncomms4930 PMC4279128 24852848

[B86] Liu Z. Park B.-J. Kanno A. Kameya T. (2005). The novel use of a combination of sonication and vacuum infiltration in *Agrobacterium*-mediated transformation of kidney bean (*Phaseolus vulgaris* L.) with lea gene. *Mol. Breed.* 16 189.

[B87] Lombardi-Crestana S. Azevedo M. D. Silva G. F. F. E. Pino L. E. Appezzato-Da-Gloria B. Figueira A. (2012). The tomato (*Solanum lycopersicum* cv. Micro-Tom) natural genetic variation Rg1 and the DELLA mutant procera control the competence necessary to form adventitious roots and shoots. *J. Exp. Bot.* 63 5689–5703. 10.1093/jxb/ers221 22915742 PMC3444280

[B88] Lor V. S. Starker C. G. Voytas D. F. Weiss D. Olszewski N. E. (2014). Targeted mutagenesis of the tomato PROCERA gene using transcription activator-like effector nucleases. *Plant Physiol.* 166 1288–1291. 10.1104/pp.114.247593 25217528 PMC4226374

[B89] Macas J. Neumann P. Navratilova A. (2007). Repetitive DNA in the pea (*Pisum sativum* L.) genome: comprehensive characterization using 454 sequencing and comparison to soybean and *Medicago truncatula*. *BMC Genomics* 8:427 10.1186/1471-2164-8-427 PMC2206039 18031571

[B90] Maggioni L. (2004). “Conservation and use of vegetable genetic resources: a european perspective,” in *Proceedings of the International Society for Horticultural Science (ISHS)* Leuven 13–30. 10.17660/actahortic.2004.637.1

[B91] Mahfouz M. M. Cardi T. Neal Stewart C. Jr (2016). Next-generation precision genome engineering and plant biotechnology. *Plant Cell Rep.* 35 1397–1399. 10.1007/s00299-016-2009-8 27271686

[B92] Manamohan M. Prakash N. Sharath Chandra G. (2011). “Cucurbits,” in *Advances in Horticulture Biotechnology* eds Nirmal K. Babu Singh H. P. Parthasarathy V. A. (New Delhi: Westville Publishing House) 227–252.

[B93] Matvieieva N. A. (2015). *Agrobacterium*-mediated transformation of compositae plants. I. Construction of transgenic plants and “hairy” roots with new properties. *Biotechnol. Acta* 8 19–31. 10.15407/biotech8.01.019

[B94] Menin B. Moglia A. Comino C. Lanteri S. Van Herpen T. W. J. M. Beekwilder J. (2012). “In vitro callogenesis and *Agrobacterium*-mediated transformation of globe artichoke,” in *Proceedings of the International Society for Horticultural Science (ISHS)* Leuven 267–271. 10.17660/actahortic.2012.961.34

[B95] Michael T. P. VanBuren R. (2015). Progress, challenges and the future of crop genomes. *Curr. Opin. Plant Biol.* 24 71–81. 10.1016/j.pbi.2015.02.002 25703261

[B96] Mikschofsky H. Broer I. (2012). Feasibility of *Pisum sativum* as an expression system for pharmaceuticals. *Transgenic Res.* 21 715–724. 10.1007/s11248-011-9573-z 22057506

[B97] Milojević J. Tubić L. Nolić V. Mitić N. Ćalić-Dragosavac D. Vinterhalter B. (2012). Hygromycin promotes somatic embryogenesis in spinach. *Plant Cell Tissue Organ Cult.* 109 573–579. 10.1007/s11248-011-9573-z

[B98] Nadolska-Orczyk A. Malepszy S. (1989). In vitro culture of *Cucumis sativus* L. *Theor. Appl. Genet.* 78 836–840. 10.1007/BF00266667 24226015

[B99] Nanasato Y. Okuzaki A. Tabei Y. (2013). Improving the transformation efficiency of *Cucurbita* species: factors and strategy for practical application. *Plant Biotechnol.* 30 287–294. 10.5511/plantbiotechnology.13.0331a

[B100] Nguyen A. H. Hodgson L. M. Erskine W. Barker S. J. (2016). An approach to overcoming regeneration recalcitrance in genetic transformation of lupins and other legumes. *Plant Cell Tissue Organ Cult.* 127 623–635. 10.1007/s11240-016-1087-1

[B101] Nguyen Q. V. Boo K. H. Sun H. J. Cao D. V. Lee D. Ko S. H. (2013a). Evaluation of factors influencing *Agrobacterium*-mediated spinach transformation and transformant selection by EGFP fluorescence under low-selective pressure. *In vitro Cell. Dev. Biol. Plant* 49 498–509. 10.1007/s11627-013-9534-8

[B102] Nguyen Q. V. Sun H. J. Boo K. H. Lee D. Lee J.-H. Lim P. O. (2013b). Effect of plant growth regulator combination and culture period on in vitro regeneration of spinach (*Spinacia oleracea* L.). *Plant Biotechnol. Rep.* 7 99–108. 10.1007/s11816-012-0242-3

[B103] Nimmakayala P. Abburi V. L. Saminathan T. Almeida A. Davenport B. Davidson J. (2016a). Genome-wide divergence and linkage disequilibrium analyses for *Capsicum baccatum* revealed by genome-anchored single nucleotide polymorphisms. *Front. Plant Sci.* 7:1646 10.3389/fpls.2016.01646 PMC5093146 27857720

[B104] Nimmakayala P. Levi A. Abburi L. Abburi V. L. Tomason Y. R. Saminathan T. (2014). Single nucleotide polymorphisms generated by genotyping by sequencing to characterize genome-wide diversity, linkage disequilibrium, and selective sweeps in cultivated watermelon. *BMC Genomics* 15:767 10.1186/1471-2164-15-767 PMC4246513 25196513

[B105] Nimmakayala P. Tomason Y. R. Abburi V. L. Alvarado A. Saminathan T. Vajja V. G. (2016b). Genome-wide differentiation of various melon horticultural groups for use in GWAS for fruit firmness and construction of a high resolution genetic map. *Front. Plant Sci.* 7:1437. 10.3389/fpls.2016.01437 PMC5031849 27713759

[B106] Nogue F. Mara K. Collonnier C. Casacuberta J. M. (2016). Genome engineering and plant breeding: impact on trait discovery and development. *Plant Cell Rep.* 35 1475–1486. 10.1007/s00299-016-1993-z 27193593 PMC4903109

[B107] O’Sullivan D. M. Angra D. (2016). Advances in faba bean genetics and genomics. *Front. Genet.* 7:150 10.3389/fgene.2016.00150 PMC4993074 27597858

[B108] Park J.-I. Ahmed N. U. Kim H.-R. Nou I.-S. (2012). Advances in in vitro culture of the Brassicaceae crop plants. *J. Plant Biotechnol.* 39 13–22. 10.1007/978-1-4939-1695-5_23

[B109] Perseguini J. M. Oblessuc P. R. Rosa J. R. Gomes K. A. Chiorato A. F. Carbonell S. A. (2016). Genome-wide association studies of anthracnose and angular leaf spot resistance in common bean (*Phaseolus vulgaris* L.). *PLoS ONE* 11:e0150506 10.1371/journal.pone.0150506 PMC4773255 26930078

[B110] Piatek A. Mahfouz M. M. (2016). Targeted genome regulation via synthetic programmable transcriptional regulators. *Crit. Rev. Biotechnol.* 10.3109/07388551.2016.1165180 [Epub ahead of print]. 27093352

[B111] Pino L. E. Lombardi-Crestana S. Azevedo M. S. Scotton D. C. Borgo L. Quecini V. (2010). The Rg1 allele as a valuable tool for genetic transformation of the tomato ‘Micro-Tom’ model system. *Plant Methods* 6:23 10.1186/1746-4811-6-23 PMC2958934 20929550

[B112] Portis E. Cericola F. Barchi L. Toppino L. Acciarri N. Pulcini L. (2015). Association mapping for fruit, plant and leaf morphology traits in eggplant. *PLoS ONE* 10:e0135200 10.1371/journal.pone.0135200 PMC4540451 26284782

[B113] Pulianmackal A. J. Kareem A. V. Durgaprasad K. Trivedi Z. B. Prasad K. (2014). Competence and regulatory interactions during regeneration in plants. *Front. Plant Sci.* 5:142 10.3389/fpls.2014.00142 PMC3990048 24782880

[B114] Punja Z. K. Jayaraj J. Wally O. (2007). “Carrot,” in *Biotechnology in Agriculture and Forestry. Transgenic Crops IV* eds Pua E. C. Davey M. R. (Heidelberg: Springer-Verlag) 277–294. 10.1007/978-3-540-36752-9_15

[B115] Qin C. Yu C. Shen Y. Fang X. Chen L. Min J. (2014). Whole-genome sequencing of cultivated and wild peppers provides insights into *Capsicum* domestication and specialization. *Proc. Natl. Acad. Sci. U.S.A.* 111 5135–5140. 10.1073/pnas.1400975111 24591624 PMC3986200

[B116] Ranc N. Munos S. Xu J. Le Paslier M. C. Chauveau A. Bounon R. (2012). Genome-wide association mapping in tomato (*Solanum lycopersicum*) is possible using genome admixture of *Solanum lycopersicum* var. *cerasiforme*. *G3 (Bethesda)* 2 853–864. 10.1534/g3.112.002667 22908034 PMC3411241

[B117] Rani R. Yadav P. Barbadikar K. M. Baliyan N. Malhotra E. V. Singh B. K. (2016). CRISPR/Cas9: a promising way to exploit genetic variation in plants. *Biotechnol. Lett.* 38 1991–2006. 10.1007/s10529-016-2195-z 27571968

[B118] Ron M. Kajala K. Pauluzzi G. Wang D. Reynoso M. A. Zumstein K. (2014). Hairy root transformation using *Agrobacterium* rhizogenes as a tool for exploring cell type-specific gene expression and function using tomato as a model. *Plant Physiol.* 166 455–469. 10.1104/pp.114.239392 24868032 PMC4213079

[B119] Rotino G. L. Sala T. Toppino L. (2014). “Eggplant,” in *Alien Gene Transfer in Crop Plants* 1st Edn Vol. 2 eds Pratap A. Kumar J. (New York, NY: Springer-Verlag).

[B120] Ruggieri V. Francese G. Sacco A. D’alessandro A. Rigano M. M. Parisi M. (2014). An association mapping approach to identify favourable alleles for tomato fruit quality breeding. *BMC Plant Biol.* 14:337 10.1186/s12870-014-0337-9 PMC4266912 25465385

[B121] Sauvage C. Segura V. Bauchet G. Stevens R. Do P. T. Nikoloski Z. (2014). Genome-wide association in tomato reveals 44 candidate loci for fruit metabolic traits. *Plant Physiol.* 165 1120–1132. 10.1104/pp.114.241521 24894148 PMC4081326

[B122] Saxena S. N. Kothari P. Rathore S. S. Ishan-Ulla-Khan and Saxena R. (2012). Organogenesis in fennel (*Foeniculum vulgare* Mill.). *Int. J. Seed Spices* 2 1–4.

[B123] Scaglione D. Lanteri S. Acquadro A. Lai Z. Knapp S. J. Rieseberg L. (2012). Large-scale transcriptome characterization and mass discovery of SNPs in globe artichoke and its related taxa. *Plant Biotechnol. J.* 10 956–969. 10.1111/j.1467-7652.2012.00725.x 22849342

[B124] Scaglione D. Reyes-Chin-Wo S. Acquadro A. Froenicke L. Portis E. Beitel C. (2016). The genome sequence of the outbreeding globe artichoke constructed de novo incorporating a phase-aware low-pass sequencing strategy of F1 progeny. *Sci. Rep.* 6:19427 10.1038/srep19427 PMC4726258 26786968

[B125] Schinkel H. Schillberg S. (2016). Genome editing: intellectual property and product development in plant biotechnology. *Plant Cell Rep.* 35 1487–1491. 10.1007/s00299-016-1988-9 27146974

[B126] Schmutz J. Mcclean P. E. Mamidi S. Wu G. A. Cannon S. B. Grimwood J. (2014). A reference genome for common bean and genome-wide analysis of dual domestications. *Nat. Genet.* 46 707–713. 10.1038/ng.3008 24908249 PMC7048698

[B127] Shirasawa K. Fukuoka H. Matsunaga H. Kobayashi Y. Kobayashi I. Hirakawa H. (2013). Genome-wide association studies using single nucleotide polymorphism markers developed by re-sequencing of the genomes of cultivated tomato. *DNA Res.* 20 593–603. 10.1093/dnares/dst033 23903436 PMC3859326

[B128] Silva Dias J. (2014). Guiding strategies for breeding vegetable cultivars. *Agric. Sci.* 5 9–32. 10.4236/as.2014.51002

[B129] Solís-Ramos L. Y. González-Estrada T. Nahuath-Dzib S. Zapata-Rodriguez L. C. Castaño E. (2009). Overexpression of WUSCHEL in *C. chinense* causes ectopic morphogenesis. *Plant Cell Tissue Organ Cult.* 96 279–287. 10.1007/s11240-008-9485-7

[B130] Somers D. A. Samac D. A. Olhoft P. M. (2003). Recent advances in legume transformation. *Plant Physiol.* 131 892–899. 10.1104/pp.102.017681 12644642 PMC1540289

[B131] Song D. Han Q. Dong Z. He Z. (2014). Genetic transformation of lettuce (*Lactuca sativa*): a review. *Afr. J. Biotechnol.* 13 1686–1693. 10.5897/AJB2014.13651

[B132] Song G.-Q. Loskutov A. V. Sink K. C. (2007). Highly efficient *Agrobacterium tumefaciens*-mediated transformation of celery (*Apium graveolens* L.) through somatic embryogenesis. *Plant Cell Tissue Organ Cult.* 88 193–200. 10.1007/s11240-006-9190-3

[B133] Soyk S. Muller N. A. Park S. J. Schmalenbach I. Jiang K. Hayama R. (2017). Variation in the flowering gene SELF PRUNING 5G promotes day-neutrality and early yield in tomato. *Nat. Genet.* 49 162–168. 10.1038/ng.3733 27918538

[B134] Sparrow P. A. C. Dale P. J. Irwin J. A. (2004). The use of phenotypic markers to identify *Brassica oleracea* genotypes for routine high-throughput *Agrobacterium*-mediated transformation. *Plant Cell Rep.* 23 64–70. 10.1007/s00299-004-0818-7 15197481

[B135] Sparrow P. A. C. Goldsack C. M. P. Østergaard L. (2011). “Transformation technology in the Brassicaceae,” in *Genetics and Genomics of the Brassicaceae* eds Schmidt R. Bancroft I. (New York, NY: Springer-Verlag) 505–525. 10.1007/978-1-4419-7118-0_18

[B136] Sprink T. Eriksson D. Schiemann J. Hartung F. (2016). Regulatory hurdles for genome editing: process- vs. product-based approaches in different regulatory contexts. *Plant Cell Rep.* 35 1493–1506. 10.1007/s00299-016-1990-2 27142995 PMC4903111

[B137] Steinert J. Schiml S. Puchta H. (2016). Homology-based double-strand break-induced genome engineering in plants. *Plant Cell Rep.* 35 1429–1438. 10.1007/s00299-016-1981-3 27084537

[B138] Sun K. Wolters A. M. Vossen J. H. Rouwet M. E. Loonen A. E. Jacobsen E. (2016). Silencing of six susceptibility genes results in potato late blight resistance. *Transgenic Res.* 25 731–742. 10.1007/s11248-016-9964-2 27233778 PMC5023794

[B139] Sun X. Zhou S. Meng F. Liu S. (2012). De novo assembly and characterization of the garlic (*Allium sativum*) bud transcriptome by Illumina sequencing. *Plant Cell Rep.* 31 1823–1828. 10.1007/s00299-012-1295-z 22684307

[B140] Svabova L. Smykal P. Griga M. Ondrej V. (2005). *Agrobacterium*-mediated transformation of *Pisum sativum* in vitro and in vivo. *Biol. Plant.* 49 361–370. 10.1007/s10535-005-0009-6

[B141] Taranto F. D’agostino N. Greco B. Cardi T. Tripodi P. (2016). Genome-wide SNP discovery and population structure analysis in pepper (*Capsicum annuum*) using genotype-by-sequencing. *BMC Genomics* 17:943 10.1186/s12864-016-3297-7 PMC5117568 27871227

[B142] Terracciano I. Cantarella C. D’agostino N. (2016). “Hybridization-based enrichment and next generation sequencing to explore genetic diversity in plants,” in *Dynamics of Mathematical Models in Biology* eds Rogato A. Zazzu V. Guarracino M. R. (Cham: Springer International Publishing).

[B143] Timko M. P. Rushton P. J. Laudeman T. W. Bokowiec M. T. Chipumuro E. Cheung F. (2008). Sequencing and analysis of the gene-rich space of cowpea. *BMC Genomics* 9:103 10.1186/1471-2164-9-103 PMC2279124 18304330

[B144] Tomato Genome Consortium (2012). The tomato genome sequence provides insights into fleshy fruit evolution. *Nature* 485 635–641. 10.1038/nature11119 22660326 PMC3378239

[B145] Truco M. J. Ashrafi H. Kozik A. Van Leeuwen H. Bowers J. Reyes Chin Wo S. (2013). An ultra high-density, transcript-based, genetic map of lettuce. *G3 (Bethesda)* 3 617–631. 10.1534/g3.112.004929 23550116 PMC3618349

[B146] van Schie C. C. Takken F. L. (2014). Susceptibility genes 101: how to be a good host. *Annu. Rev. Phytopathol.* 52 551–581. 10.1146/annurev-phyto-102313-045854 25001453

[B147] Veltcheva M. Svetleva D. Petkova S. Perl A. (2005). In vitro regeneration and genetic transformation of common bean (*Phaseolus vulgaris* L.)—Problems and progress. *Sci. Hortic.* 107 2–10. 10.1016/j.scienta.2005.07.005

[B148] Vinoth A. Ravindhran R. (2015). Efficient plant regeneration of watermelon (*Citrullus lanatus* Thunb.) via somatic embryogenesis and assessment of genetic fidelity using ISSR markers. *In Vitro Cell. Dev. Biol. Plant* 52 107–115. 10.1007/s11627-015-9731-8

[B149] Vinterhalter D. Sretenovič-Rajičič T. Vinterhalter B. Ninkovic S. (2007). Genetic transformation of *Brassica oleracea* vegetables. *Transgenic Plant J.* 1 340–355.

[B150] Vlasova A. Capella-Gutierrez S. Rendon-Anaya M. Hernandez-Onate M. Minoche A. E. Erb I. (2016). Genome and transcriptome analysis of the Mesoamerican common bean and the role of gene duplications in establishing tissue and temporal specialization of genes. *Genome Biol.* 17 32 10.1186/s13059-016-0883-6 PMC4766624 26911872

[B151] Voytas D. F. (2013). Plant genome engineering with sequence-specific nucleases. *Annu. Rev. Plant Biol.* 64 327–350. 10.1146/annurev-arplant-042811-105552 23451779

[B152] Wang S.-L. Ku S. S. Ye X.-G. He C.-F. Kwon S. Y. Choi P. S. (2015). Current status of genetic transformation technology developed in cucumber (*Cucumis sativus* L.). *J. Integr. Agric.* 14 469–482. 10.1016/S2095-3119(14)60899-6

[B153] Wang X. Wang H. Wang J. Sun R. Wu J. Liu S. (2011). The genome of the mesopolyploid crop species *Brassica rapa*. *Nat. Genet.* 43 1035–1039. 10.1038/ng.919 21873998

[B154] Wang Y. Yau Y. Y. Perkins-Balding D. Thomson J. G. (2011). Recombinase technology: applications and possibilities. *Plant Cell Rep.* 30 267–285. 10.1007/s00299-010-0938-1 20972794 PMC3036822

[B155] Wȩdzony M. Szechyńska-Hebda M. Żur I. Dubas E. Krzewska M. (2014). “Tissue culture and regeneration: a prerequisite for alien gene transfer,” in *Alien Gene Transfer in Crop Plants: Innovations, Methods and Risk Assessment* Vol. 1 eds Pratap A. Kumar J. (New York, NY: Springer) 43–75.

[B156] Woo J. W. Kim J. Kwon S. I. Corvalan C. Cho S. W. Kim H. (2015). DNA-free genome editing in plants with preassembled CRISPR-Cas9 ribonucleoproteins. *Nat. Biotechnol.* 33 1162–1164. 10.1038/nbt.3389 26479191

[B157] Xu L. Huang H. (2014). “Chapter One – Genetic and epigenetic controls of plant regeneration,” in *Current Topics in Developmental Biology* ed. Brigitte G. (Cambridge, MA: Academic Press) 1–33. 10.1016/B978-0-12-391498-9.00009-7 24512704

[B158] Yamamoto M. Nishio T. (2014). Commonalities and differences between *Brassica* and *Arabidopsis* self-incompatibility. *Hortic. Res.* 1 14054 10.1038/hortres.2014.54 PMC4596330 26504553

[B159] Zaidi S. Tashkandi M. Mansoor S. Mahfouz M. (2016). Engineering plant immunity: using CRISPR/Cas9 to generate virus resistance. *Front. Plant Sci.* 7:1673 10.3389/fpls.2016.01673 PMC5099147 27877187

[B160] Zhang H. Gao P. Wang X. Luan F. (2014). An improved method of *Agrobacterium tumefaciens*-mediated genetic transformation system of melon (*Cucumis melo* L.). *J. Plant Biochem. Biotechnol.* 23 278–283. 10.1007/s13562-013-0211-0

[B161] Zhang J. Zhao J. Xu Y. Liang J. Chang P. Yan F. (2015). Genome-wide association mapping for tomato volatiles positively contributing to tomato flavor. *Front. Plant Sci.* 6:1042 10.3389/fpls.2015.01042 PMC4661238 26640472

[B162] Zhang N. Huang X. Bao Y. Wang B. Liu L. Dai L. (2015). Genome-wide identification and expression profiling of WUSCHEL-related homeobox (WOX) genes during adventitious shoot regeneration of watermelon (*Citrullus lanatus*). *Acta Physiol. Plant.* 37 224 10.1007/s11738-015-1964-y

[B163] Zheng S.-J. Henken B. Kyun Ahn Y. Krens F. A. Kik C. (2004). The development of a reproducible *Agrobacterium tumefaciens* transformation system for garlic (*Allium sativum* L.) and the production of transgenic garlic resistant to beet armyworm (*Spodoptera exigua* Hübner). *Mol. Breed.* 14 293–307. 10.1023/B:MOLB.0000047775.83715.b5

[B164] Zheng S.-J. Khrustaleva L. Henken B. Sofiari E. Jacobsen E. Kik C. (2001). *Agrobacterium tumefaciens*-mediated transformation of *Allium cepa* L.: the production of transgenic onions and shallots. *Mol. Breed.* 7 101–115. 10.1023/A:1011348229189

[B165] Zsögön A. Cermak T. Voytas D. Peres L. E. P. (2017). Genome editing as a tool to achieve the crop ideotype and de novo domestication of wild relatives: case study in tomato. *Plant Sci.* 256 120–130. 10.1016/j.plantsci.2016.12.012 28167025

